# Exploring Couples’ Processes of Change in the Context of SASA!, a Violence Against Women and HIV Prevention Intervention in Uganda

**DOI:** 10.1007/s11121-016-0716-6

**Published:** 2016-09-29

**Authors:** Elizabeth Starmann, Martine Collumbien, Nambusi Kyegombe, Karen Devries, Lori Michau, Tina Musuya, Charlotte Watts, Lori Heise

**Affiliations:** 1 London School of Hygiene & Tropical Medicine, London, UK; 2 Raising Voices, Kampala, Uganda; 3 Center for Domestic Violence Prevention, Kampala, Uganda

**Keywords:** Partner violence, Violence against women, Relationship change, Community mobilisation, SASA!

## Abstract

There is now a growing body of research indicating that prevention interventions can reduce intimate partner violence (IPV); much less is known, however, about how couples exposed to these interventions experience the change process, particularly in low-income countries. Understanding the dynamic process that brings about the cessation of IPV is essential for understanding how interventions work (or don’t) to reduce IPV. This study aimed to provide a better understanding of how couples’ involvement with SASA!—a violence against women and HIV-related community mobilisation intervention developed by Raising Voices in Uganda—influenced processes of change in relationships. Qualitative data were collected from each partner in separate in-depth interviews following the intervention. Dyadic analysis was conducted using framework analysis methods. Study findings suggest that engagement with SASA! contributed to varied experiences and degrees of change at the individual and relationship levels. Reflection around healthy relationships and communication skills learned through SASA! activities or community activists led to more positive interaction among many couples, which reduced conflict and IPV. This nurtured a growing trust and respect between many partners, facilitating change in longstanding conflicts and generating greater intimacy and love as well as increased partnership among couples to manage economic challenges. This study draws attention to the value of researching and working with both women, men and couples to prevent IPV and suggests IPV prevention interventions may benefit from the inclusion of relationship skills building and support within the context of community mobilisation interventions.

## Introduction

Violence against women (VAW) is an abuse of women’s rights, with significant impacts on women’s health (Devries et al. 2013; Ellsberg et al. 2008), including increased vulnerability to HIV (Kouyoumdjian et al. 2013; UNAIDS 2012). Intimate partner violence (IPV) is the most common form of violence against women, with 30 % of women globally experiencing it during their lifetime (Devries et al. 2013). A multitude of factors influence partner violence beyond the individual level, and prevention programming has slowly evolved to include interventions (e.g. community mobilisation approaches) that reach across the relational, community and institutional levels of the social ecology (Heise 2011).

Efforts to address VAW have expanded from a focus on assisting survivors (e.g. shelters, legal and psychosocial support) to include prevention programming aimed at stopping violence before it starts (primary prevention), preventing its re-occurrence (secondary prevention) and mitigating its impact (tertiary prevention), as well as comprehensive programmes aimed at all three (Krug and Dahlberg 2002). For example, multi-level community mobilisation approaches engage a range of individuals and groups across the ecological model over time, using different strategies aimed at fostering critical reflection and individual and collective action to prevent IPV. There is now a growing body of evidence on the impact of prevention interventions in different contexts. Rigorous trials in Sub-Saharan Africa suggest community mobilisation and reflective strategies work to prevent IPV (Hossain et al. 2014; Jewkes et al. 2008; Wagman et al. 2015); however, there is less clarity on “how they work”. A critical gap remains—especially in low-income countries—on how couples with a history of IPV actually change following exposure to multi-level prevention interventions. Understanding desistance—the dynamic process that supports and brings about the cessation of IPV perpetration—within the context of multi-level prevention is essential for understanding how interventions work (or don’t) to inform prevention efforts (Walker et al. 2013). This is particularly important in contexts with a high prevalence of IPV, where a larger portion of the population are in relationships with previous or ongoing violence.

To date, the theoretical frameworks in the field of IPV prevention used to inform intervention designs have mainly encompassed risk factors in the aetiology of IPV (e.g. the ecological model) (Heise 2011) and behaviour change processes at the individual (e.g. transtheoretical and health belief models), interpersonal (e.g. social cognitive theory) and community levels (e.g. diffusion of innovations theory and community mobilisation theory) (DiClemente et al. 2011; Glanz and Bishop 2010). A recent review of the literature on desistance from IPV found only 15 eligible studies from 1980 to 2011 and no single theory explaining desistance was identified (Walker et al. 2013). The psychology and sociology literature on gender and power, relationship education, family process and couples therapy offers more insight. It provides a useful evidence base on key constructs and processes that influence relationship quality and partner violence including relationship equality (Krishnan et al. 2012), effective communication (Overall et al. 2009), self-regulation (Hira and Overall 2011), shared investment (Fincham et al. 2007) and power (Knudson-Martin 2013; Rabin 1994). Power in the relationship context refers to the capacity or ability of one partner to change their partner’s feelings, thoughts or behaviours to align with their own desired preferences, combined with the ability to resist their partner’s influence attempts (Simpson et al. 2015). This literature also offers insight into what motivates individuals to make difficult relationship changes. For example, sociologists have suggested that hope is the combination of “waypower”—the pathway (e.g. new relationship skills) towards a goal—and “willpower”—the motivation to move along the pathway towards the desired goal (e.g. improved relationship quality/cessation of IPV) (Snyder 1994; Snyder et al. 2000). While the existing literature is mainly from the global North, there is a growing body of research in African contexts linking power, relationship quality and IPV (Conroy 2014; Jewkes et al. 2010; Krishnan et al. 2012).

Relationship dynamics and change processes are also influenced by community and societal factors. Benjamin and Sullivan’s (1999) model of change in marital relationships uniquely acknowledges the multiple levels of influence at play, emphasising the interconnected relationships between intimacy, power, resources and their material expression. In their study of couples in the United States, they found change is centred on the interplay of gender consciousness, relational resources (a combination of emotional and interpersonal resources and skills partners bring to relationships) and, to a lesser degree, material resources (income, access to financial resources). Gender consciousness is considered a continuum from general awareness to knowledge of gender specific rights awarded in a given system, to recognition of how one reproduces them in social interactions, to challenging that system to change it (Gerson and Peiss 1985). This incorporation of multi-level influences makes the model a useful guide for examining desistance from partner violence.

The present study seeks to understand the processes that led to change in the relationships of couples exposed to SASA!, a multi-level community mobilisation intervention aimed at preventing VAW and HIV. It forms part of the SASA! study, a multidisciplinary evaluation comprising a cross-sectional cluster randomised control trial (RCT) (Abramsky et al. 2014), qualitative studies, a process evaluation and a costing study (Michaels-Igbokwe et al. 2016). The RCT showed the intervention to be associated with lower acceptability of IPV, as well as reductions in women’s experiences of IPV—past year experience of all types of IPV was lower in intervention compared to control communities, with statistically significant effects observed for past year experience of high intensity emotional aggression and controlling behaviours, and cessation of physical, sexual and emotional IPV where it was previously occurring (Abramsky et al. 2016). The main qualitative evaluation found SASA! helped foster an environment of non-tolerance of violence by decreasing the acceptability of violence against women and increasing individuals’ skills and sense of responsibility to act to prevent it (Kyegombe et al. 2014b). It was also found the intervention developed and strengthened community-based structures to support ongoing activism to prevent IPV. Secondary analyses further suggest the intervention impacted HIV-related risk behaviours (Kyegombe et al. 2014a).

This couples study, in turn, provides a dyadic examination of the change processes of couples who were both exposed to the intervention and experienced a cessation in IPV. It is not intended to examine the effectiveness of SASA!; rather it aims to understand how some relationships improved and violence waned. It draws on data collected from the perspectives of both partners, offering a rich understanding of the relationship dynamics and change processes. The practice of interviewing both members of couples is surprisingly uncommon in the field of violence prevention and there are calls in the literature for more dyad (couple) research (Davis et al. 2012; Johnson 2010; Wadsworth and Markman 2012). The initial couples study design was conceptualised around the transtheoretical model’s stages of change (Prochaska et al. 2008) (also used in SASA!’s theory of change) and the data analysis then drew on the marital change model (Benjamin and Sullivan 1999) and relational concepts from the psychology and sociology literature. This is—to our knowledge—the only study in a low-income context examining desistance and relational change among couples resulting from exposure to a multi-level prevention intervention.

## Study Setting and Intervention

The SASA! intervention (Michau 2008), detailed in Box 1, was designed by Raising Voices and implemented in Kampala, Uganda, by the Center for Domestic Violence Prevention (CEDOVIP). The SASA! study was conducted in eight high-density, impoverished communities in Kampala, Uganda. There was a 6-month interruption in programming due to political unrest and follow-up was extended to 4 years after baseline to allow the full intervention to be delivered as intended. Mobility in the study setting was high with a large proportion of people who had migrated from other parts of the country to Kampala for employment. HIV prevalence remains high in Kampala with 9.5 % of women and 4.1 % of men aged 15–49 estimated to be HIV positive (Uganda Ministry of Health and ICF International 2012). Levels of IPV in Uganda are high, with 45 % of ever-married women aged 15–49 reporting having experienced physical and/or sexual violence by an intimate partner at some point in their lives (Uganda Bureau of Statistics and ICF International 2012). Partner violence is closely linked to the changing gender roles and expectations around relationships in Uganda, as well as alcohol use and multiple sexual partners (Karamagi et al. 2006; Koenig et al. 2003). Hegemonic masculinity—the ideal form of masculinity at a given time and place that subordinates women and some men (Connell and Messerschmidt 2005)—also plays a role. For example, men’s traditional role as provider gives them authority over women and financial decisions in Uganda (Wyrod 2008). This role is under threat due to poverty, and in similar settings of economic hardship, it is suggested partner violence may be men’s response to the loss of identity and self-esteem attached to the provider role (Silberschmidt 2001).

**Table:** 

**Box 1: SASA! Intervention** *SASA!* is a community mobilisation approach for preventing VAW and HIV. It is designed for catalysing community-led change of norms and behaviours that perpetuate gender inequality, violence and increased HIV vulnerability for women. SASA! means “Now” in Kiswahili and is an acronym for the four phases of the approach—Start, Awareness, Support, Action. In the Start phase, an organisation using *SASA!* begins by orienting staff to the approach and key concepts of power. They then select an equal number of female and male community activists (CAs)—regular people in the community interested in issues of violence, power and rights—and similarly select institutional activists, for example, from police, health care, local government and faith-based groups. All activists are introduced to the new ways of thinking about power and power imbalances in their own lives and within the community, and are mentored in the *SASA!* approach. With the support of staff, the activists then take the lead as the approach moves forward into the Awareness, Support and Action phases. In these phases, the community activists lead informal, benefits-based activities within their existing social networks—fostering open discussions, critical thinking and supportive person-to-person and public activism among their families, friends, colleagues and neighbours. Together, they introduce the community and its institutions to the new concepts of power, encouraging a gendered analysis of power imbalances through four strategies: Local Activism, Media and Advocacy, Communication Materials, and Training. This includes a focus on core relationship values such as love, respect, trust and joint decision making. The combination of these strategies ensures that community members are exposed to *SASA!* ideas repeatedly and in diverse ways within the course of their daily lives, from people they know and trust as well as from more formal sources within the community. Each phase builds on the other and addresses a different concept of power, with an increasing number of individuals and groups involved, strengthening a critical mass committed and able to create social norm change (Raising Voices 2015).	

## Methodology

Qualitative methods were used to examine the processes of change and relationship trajectories of couples in which at least one partner had been exposed to SASA!. Semi-structured interviews were conducted with each partner separately to obtain a more comprehensive picture of the relationship from both perspectives (Eisikovits and Koren 2010; Hertz 1995). Participants were sampled purposively from the RCT survey data collected in 2012 at follow-up (apart from one sampled via snowballing from another interview). RCT participants that agreed to be contacted again were sampled using the following criteria: in current relationship since 2010 or before; IPV reported before the last 12 months, but not in the last 12 months; exposure to SASA! (any intensity); and, reported positive change in relationship since becoming involved in SASA!. For ethical reasons, we excluded couples with ongoing IPV to ensure participants’ safety (Watts et al. 1999). Initial efforts to recruit couples through contacting female RCT participants yielded only two couples. While women consented to have their partner interviewed, the men proved reluctant. Having not been interviewed during the RCT (only one partner per household was interviewed), they were sceptical of the researchers’ interview requests (e.g. some thought they might be debt collectors). Therefore, eight couples were recruited through male RCT participants with further precautions taken to ensure their female partners were not pressured into participating. Twenty individual interviews (ten female, ten male) were conducted between June and October 2012 with partners from ten heterosexual couples sampled across the four intervention communities.

The interview guide was developed and translated from English to Luganda in consultation with staff from Raising Voices and CEDOVIP, and piloted and finalised with the research team. The guide starts with general questions about the participant’s relationship and any changes they have observed. This allowed participants to first mention SASA! of their own accord as well as attribute any changes in their relationship to it (or not). Later in the guide, there are more specific questions and probes about SASA! exposure and how it impacted their relationship. Given the challenges of recalling relationship events over time (Chang et al. 2006), a participatory timeline tool was created by the first author to help participants map out when different life and relationship events happened (including the timing of their exposure to SASA!).

The research team comprised two female and two male SASA! research assistants trained in conducting IPV research and qualitative research techniques. The WHO protocol for interviewing women on VAW was observed (Watts et al. 1999) to ensure the safety of participants. After both partners were contacted and agreed to the interview, a male and female research team went to their home and partners were interviewed separately, but concurrently in a private place of their choice by the same-sex researcher. Each participant gave individual written informed consent to be interviewed and audio recorded. Recordings were transcribed and translated using a single-stage transcription process and regular fidelity checks conducted to ensure quality. Couples were numbered with partners indicated by M for male, F for female (e.g. 1F, 1M) and pseudonyms used to protect confidentiality.

The data was analysed by the first author using framework analysis and couple timeline maps were built from each partner’s transcript. Framework analysis is a matrix-based method that permits the researcher to systematically organise “raw” data under thematic framework matrices for continuous analysis across themes and cases, while retaining links to the original data (Ritchie et al. 2003).

Data analysis was iterative and began during post-interview debrief sessions with the tool slightly modified as new themes came up. Paper transcripts were then open coded to allow the data to speak for itself (Green and Thorogood 2009). The most prevalent codes were organised into a coding framework or index with main themes and sub topics. Transcripts were then uploaded into NVivo 10 software (QSR International Pty Ltd 2012) and coded using the coding framework. To assist the dyadic analysis of the data, couple summaries and a joint timeline map of the sequence of relationship events was built for each couple from the transcripts and the timeline tool used during the interviews.

The maps offered a visual means to observe patterns and together with the data indexing process, common themes in relationship trajectories and change emerged. Thematic framework matrices were then auto-generated in NVivo. Each matrix contained all “raw” data coded under each theme and sub topic organised by case. Next, the coded text for each case was summarised and manually reduced. This process helped ensure the data did not lose the context or content when pulled from a transcript (Gale et al. 2013).

A descriptive analysis then further refined the data into categories under broader classifications, followed by associative analyses to detect patterns between themes and across different cases. This included categorising the health of couples’ relationships (constructed based on the presence of different forms and severity of violence and the degree to which the couple balanced power and communicated) prior to and after SASA! exposure. At this stage in the analysis, we engaged concepts and theory from the wider relationship, psychology and family process literature (e.g. gender consciousness and relational resources from Benjamin and Sullivan’s marital change model) to help understand the salient themes observed in couples’ relationships and processes of change. Finally, explanations for the associations were developed by moving back and forth between the matrices, transcripts, timeline maps and literature.

## Findings

The majority of couples were in their 30s and 40s with relationships spanning 2 ½–25 years (Table 1). Couple 9 was found to be separated at the time of the interview, but the decision was made to include them as their exposure to SASA! had brought about positive changes in their relationship despite their separation. Couples had at least one child together and many had additional children from previous relationships. The intensity and type of exposure to SASA! varied among participants. In seven of the ten couples, both partners had been exposed to either SASA! activities or had direct support from a community activist, with only two female participants (Esther and Mary) reporting no exposure at all.

**Table 1:** Overview of couples sampled

Couple #	Name (pseudonym)	Relationship duration (years)	# Children together (# previous children by male (M) or female (F))
1	Janice and Joseph	12	5
2	Stella and Henry	23	4 (M-1)
3	Milly and Andrew	25	4 (M-1)
4	Patience and Peter	8	4 (F-2)
5	Esther and Frank	8	3 (F-5)
6	Jean and Charles	3	1 (M-multiple)
7	Sarah and Paul	16	6
8	Mary and Robert	2.5	1 (F-1)
9	Betty and Martin	18 (separated)	2 (M-1)
10	Florence and Isaac	4.5	1 (M-3)

The overall health of participants’ relationships prior to SASA! exposure was generally poor with some variation along a spectrum (this is expected as we purposely sampled couples reporting IPV before the last 12 months). Some couples (1, 5, 8, 9) did not spontaneously report a history of physical or sexual IPV in the qualitative interviews even though it was a criteria for recruitment from the survey. In contrast to the survey, the qualitative tool did not ask about specific acts of IPV, and some participants may not have considered less severe forms of physical violence (e.g. pushing, shoving, slapping) to be violence. Four couples (2, 4, 7, 10) reported more severe forms of physical violence occasionally and two couples (3 and 6) rarely. All couples reported other forms of IPV (e.g. controlling behaviour and verbal abuse) as well as distrust (e.g. around income, fidelity), frequent quarrelling, and poor communication and power sharing. Conflict and different forms of abuse arose from a variety of interrelated pressures linked to personal history, socioeconomic challenges and gender role conflicts. These conflicts were significantly amplified by both poverty and rigid gender norms, for example, as couples tried to navigate the tension between fulfilling the gender roles they felt judged by, with the difficult economic realities in their context.

### Processes of Change

**Fig. 1:**
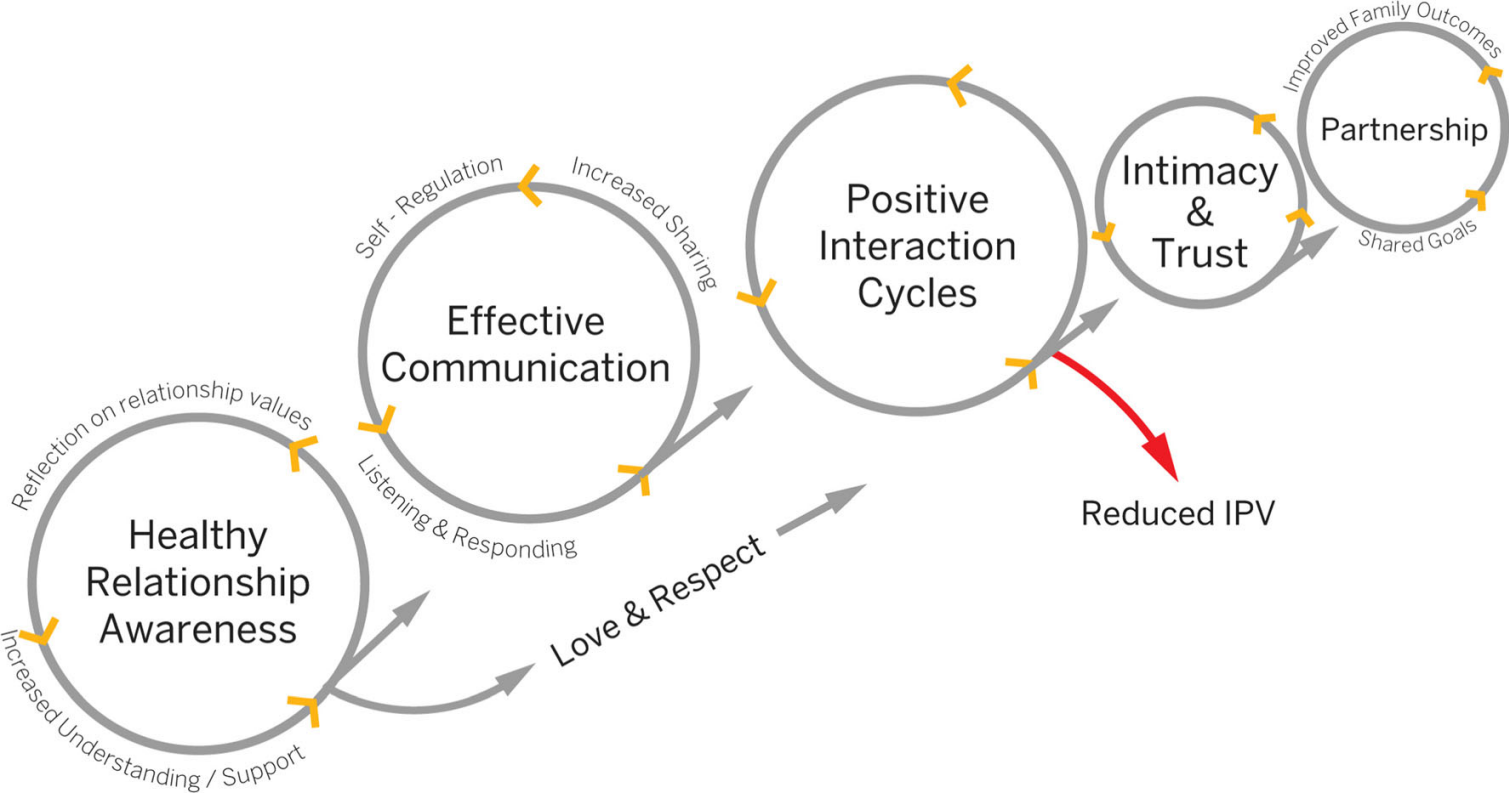
Processes of change

Engagement with SASA! by one or both members of couples resulted in a range of change processes at the individual and relational levels that contributed to the cessation of IPV (Fig. 1). To start, SASA! appeared to generate curiosity and reflection on what constitutes a healthy relationship. Many noted they never learned how to be in a healthy relationship:

[F]or me I entered marriage without any form of counselling from anyone so by attending these activities I have learnt a lot. (4F)

Through enhanced awareness of relationship values (e.g. love and respect) and gender roles, some participants began reflecting on their own and their partner’s role and how greater mutual support could result in better outcomes for their family. Shifts around this were mainly expressed as a “softening” of their or their partner’s previous relationship expectations linked to traditional gender roles. For example, “more understanding” around their husband’s struggle to provide and becoming more open to their wife working. However, shifts around gender roles proved difficult for others, in particular around the issue of women working. In some cases, this new awareness was also challenging and emotionally painful when their partner was unwilling to change. For example, Janice in couple 1 had an HIV test after seeing a SASA! drama and asked her husband to get tested. He refused repeatedly and, though she feared for her health and was deeply hurt, she felt unable to push him:

I gave up, and I dropped the issue…so we moved on…because I did not want us to get disorganised [experience distress/conflict]. (1F)

Notably, this was mostly among couples like Janice and Joseph where one or both partners had minimal exposure to SASA!.

Next, relationship skills learned from SASA! activities or CA support led to more positive interaction patterns for many couples. Self-regulation techniques featured prominently, particularly learning to “keep quiet” during heated exchanges by leaving the room or home till they “calmed down” and could discuss things. This was also the most common example participants gave of how they or their partner had changed. For example, Florence in couple 10 shared,

[Y]ou realise that it [SASA!] changes people, like a person who is hot tempered like me, I learned how to control this temper. (10F)

Her husband likewise noted,

[T]he biggest change I got was to learn to keep quiet when there are problems instead of fight… I will move around town and by the time I come back, I have a better approach. (10M)

These changes around self-regulation were valued by participants because they prevented fights from escalating, curbing verbal abuse and/or physical violence. For some, there was a new awareness that if they changed their behaviour, their partner would as well:

[W]hen you keep quiet and you calm yourself down, you will realize that she will also calm down, she will speak to herself and change. (2M)

This illustrates how partners influenced changes in each other that impacted the relationship as a whole, sometimes even when one partner had little to no exposure to SASA!.

While SASA! encourages both women and men to manage their own emotions and reactions to challenges in the relationship, some incomplete, misguided or inconsistent applications of the suggested self-regulation techniques were observed. The most common example concerned “keeping quiet” during a heated moment, but not returning to discuss the issue when calm as SASA! messages encourage. Some used “keeping quiet” or silence as a coercive way to control their partner or withdraw to avoid the issue. However, this was only noted in participants with lesser exposure such as Janice (1F) and Betty (9F) who had only attended a few activities. Others such as Mustafa (10M) were inconsistent, at times returning to discuss the issue when calm and other times not. Above he shared how he now uses “keeping quiet” as suggested, but also gave another example:

xI can decide to keep quiet for more than 3 days until she feels concerned…I don’t respond to whatever she says and I never ask for anything. It has been my very strong weapon. (10M)

This reflects the way some participants used it to withdraw from tense issues or control their partner. The benefits of only “keeping quiet” (and not discussing later) were often not clear cut as it prevented verbal and physical violence from erupting, generating better family outcomes, but also left the core issue and tensions unresolved (i.e. major disagreements, infidelity, controlling behaviour).

More effective communication through listening and responding and sharing more was another key mechanism of change. Participants reported sharing more on topics they previously avoided such as their income, spending, struggles and feelings. These efforts to communicate were successful and influenced change in the relationship when their partner in turn listened and was responsive:

[W]hatever you tell her, if you will just be ignored, then there is no reason for you to tell her. But if you see her calming down, you also start getting moved to start talking to her. (7M)

Increased listening and responding appeared to be a particularly meaningful change for many, as partners perceived they had influence and were valued—an important indicator of power shifts. These more positive interaction cycles generated greater intimacy and love.

[W]e started smiling, we started talking and discussing issues well together. (3M)

What I am most happy about is the agreeing and understanding each other…it shows love in the relationship. (4M)

Many men and women were described by their partners as shifting from being generally “tough” to “softening” and being “more understanding” and “caring” overall.

Greater communication and openness also fuelled an increase in trust and respect between many partners. This was a key or “the most important” relationship change for many perhaps because, similar to being heard, being trusted and respected indicated they had influence/power in the relationship. This facilitated change in key conflict areas. For example, some partners that were previously controlling—due to fears their partner would be unfaithful and leave them—seemed to feel more secure. Improved communication and intimacy with their partner gave them more confidence to trust and demonstrate respect in turn by trusting their partner. For example, participants reported they (or their partner) no longer “have a problem” with their partner’s whereabouts (“I may not ask at all”) or who was calling them (“he gave up that thing [argument] about the phone”). Men were also doing more to earn their partner’s trust through communicating their earnings and struggles to provide: “Now she can even believe me when I tell her that I don’t have money” (3M). Apart from showing more understanding, women in couples 2, 3 and 4 were in turn more willing to contribute their own money where they used to “hide it”.

More trust and respect contributed to improved coping, alliance and overall partnership among couples to pursue shared goals and investment. Both women and men reported they were now “planning together” and working towards the “development of the family”. For example, Andrew and Peter in couples 3 and 4 had not been working regularly for years; their narratives suggest they felt trapped in a hopeless state, drinking and gambling to escape, while their wives provided for the family. Financial pressures incited anger, distrust, verbal abuse and, at times, physically violent fights.

[S]he realised that the money I was giving her was not enough to cater for all the expenses at home so she also started working. At the time I instead got wasted and I became completely stupid moving around the village. I started gambling; playing the board games while she was busy selling blouses…I stopped bringing anything to her (sharp clap of hands). And because of that our love was reducing and we started behaving as if we did not love each other at all. (3M)

SASA! appeared to offer these men a sense of hope that things could be different at home. They reported that CA support had given them the push they needed to stop drinking/gambling, work together with their wife and actively seek work. Due to SASA! exposure, Patience also had the confidence and skills to approach Peter differently:

I learnt how to approach him with respect and this has helped so much. He had a feeling that I was disrespecting him because he was poor…I also changed tactics on how to encourage him to work by giving him examples of how other people were behaving…this really worked so much because he got a job and he is now respected in the community. (4F)

This demonstrates the finding that many couples’ change processes were nudged along by one partner making a small change (Patience changing tactics for example) that gave the other the courage to also make some changes in their behaviour without fear of losing their perceived power or influence in the relationship, generating intimacy and a gradual improvement in their relationship. These findings also point to an unanticipated impact that SASA! appears to have had on the financial situation of many families. As Florence explained,

if you have a violence free home you can improve your livelihood and you can communicate well, you can survive on the little that you have. (10F)

Through increased partnership and communication, couples’ economic situations improved to varying degrees, with only couple 1 reporting no change in this area. However, while the majority of couples experienced improvements in communication and resolving conflict, some couples (2, 5, 7, 8, 10) continued to struggle with issues they fundamentally disagreed on such as where to invest money, parenting decisions and extended family matters.

### Key Factors Influencing Change

Several factors related to the intervention and relationship characteristics appeared to influence the processes of change described and the extent of change experienced. While reported on separately here, it was frequently a combination of different factors that converged to influence or prevent change. To start, couples’ perceived need to change appeared to influence their engagement with SASA! and application of learning, facilitating change. Those reporting the more severe and frequent forms of physical and other types of violence had the most stable and fulfilling relationships among the sample post-intervention. The motivation to change came in part from being at rock bottom within their relationship and willing to try anything to improve things:

[B]y the time this violence reached this level, she had sworn to quit the relationship and go back to her parents’ home. (3M)

Couples not experiencing physical violence when exposed to SASA! reported less relationship change overall. For example, Joseph in couple 1 perceived SASA! was not relevant for him because there was no physical violence in his relationship. However, he was controlling, preventing his wife from working and refusing her requests that he test for HIV—all issues SASA! activities address.

The combination of both partners being exposed to multiple activities and CA support appeared to facilitate the most change. Such exposure was only observed among couples that experienced deep change. Couple 6 best demonstrates this. Jean reported Charles to the Local Council (LC) leader—who handles such issues (and is also trained as part of SASA!)—for not providing for the household’s needs as is expected by social and gender norms in the context. They received relationship support from the LC and CA and started attending SASA! activities. Their narratives demonstrate a growing awareness around healthy relationships from activity attendance as they came to see their part in relationship issues and make changes in communication:

[S]he listens to me, and I also listen to her.... The communication is also good, she can tell me that this is not good and I also tell her that I have not liked this. You solve the issue peacefully without a tug of war. (6M)

Through ongoing CA support, Charles reconsidered his opposition to Jean working. This eased financial pressures for both and Jean felt less dependent on Charles. Couple 3, however, deviated notably by experiencing profound change when only one partner was exposed to SASA!. In this case, change needed to come mainly from one partner as Andrew had been unemployed, drinking and gambling for over 10 years while Milly supported the family. His intensive engagement with SASA!, individual changes and his wife’s positive support transformed their relationship and family.

CA support (e.g. regularly checking in with couples/informal counselling, positive peer pressure, etc.) appeared to act as an important helping relationship, bolstering individual and couple change processes. Participants described how through a casual and balanced approach CAs were able to support both partners to discuss contentious issues and slowly make positive changes. Ongoing CA support was particularly helpful with behaviours that proved difficult to change, for example, those challenging traditional gender roles such as women working. CAs also appeared to provide a degree of accountability for the changes partners committed to. For example, after Florence reported Isaac for using violence, the LC and CA made it clear he would be held accountable for his behaviour:

[T]hey warned him, I think they all scared him…he realised that he had to change. (10F)

But, they also offered their support to Isaac during this change process such that he felt comfortable reaching out to them:

Whenever we would experience violence, he [Isaac] is the one who would call them. (10F)

This also reflects how participants valued the close and immediate relationship resource CAs provided, noting how CAs “live nearby” and would come over “that very night” when they were experiencing distress. However, while more intensive CA support was key for some couples (2, 3, 6, 10), couple 4 did not have any, but experienced deep change from only frequent activity attendance by both partners. Overall, the findings suggest absorbing and applying new ideas and skills around healthy relationships can take time and benefit from more intense exposure, in particular, CA support and having both partners involved.

## Discussion

The results demonstrate engagement with SASA! by one or both members of couples contributed to varied experiences and degrees of change at the individual and relationship levels. Relationship changes were not universal or rapid for the most part, but often uneven and slow. Overall, greater awareness of healthy relationship values (and to a lesser degree gender consciousness) and increased relational resources (communication and self-regulation skills) led to more positive interaction patterns (increased communication, mutual financial disclosure, peaceful conflict resolution) and shifts in relationship dynamics (greater trust, respect, love and intimacy). In most couples, this resulted in greater partnership and increased financial stability/material resources.

Important shifts in power dynamics were experienced by some couples through their exposure to SASA!. This is evidenced by the valued changes reported by participants in increased communication, trust, respect, intimacy and shared goals—all indicators of more balanced power and influence noted in the literature (Knudson-Martin 2013; Simpson et al. 2015). While our sample was small, a larger sample of individuals (not couples) interviewed in our qualitative study on the lived experience of SASA! reported similar changes in their relationships (Kyegombe et al. 2014a, b). This suggests prevention interventions can cultivate changes in relationship power dynamics, an important finding given the evidence that IPV is more common among couples with power imbalances (Conroy 2014; Jewkes et al. 2010). For example, a Tanzanian study found those who shared relationship power and sexual decision making with their partner were less likely to report partner violence (Krishnan et al. 2012).

So what facilitated these shifts in power/influence? To start, SASA!’s focus on core relationship values such as love, respect and trust appeared to nurture a willingness to make changes. Research on renegotiating gender roles and power dynamics in relationships has likewise shown that intimacy and love can play a powerful role in bringing about change (Deutsch 2007). For example, research in Honduras on women’s empowerment and marital change in couples concluded, “these findings hint at the power of love as a transformative force,” and highlights how “[t]he role of love and care in relationships supports feminist theories of power as capacity rather than domination” (Murphy-Graham 2010, p. 326). This points to the perhaps untapped potential for interventions to promote love and intimacy as a mechanism to achieve more balanced power in relationships and prevent IPV. Indeed, SASA! initially focused more on sharing household tasks more equally and across stereotypical gender roles as a route to improved relationships, but program staff found this instigated adversarial dynamics between partners over who did what in the household (Namy et al. 2015). Focusing more on relationship values such as respect, love and fairness was found to be more effective in creating positive relationship dynamics and collaboration instead of competition.

The concept of hope also appeared to initiate a process of change and facilitate the gradual shifts in power for some, particularly in more distressed relationships. SASA! seems to have offered individuals a pathway (the “waypower”) towards a better relationship/family life with specific small actions they could try alongside direct support from CAs in some cases. The combination of CA support and activities appeared particularly instrumental in influencing power shifts and helping couples negotiate difficult changes. Changes were then reinforced, for example, when men experienced positive outcomes such as reduced stress over being the sole provider, greater intimacy and improved family life. This suggests, as Deutsch noted, “[m]en sometimes need and want love and care from women enough to be willing to trade power for it” (2007, pp. 121–122).

Our findings yield partial support for Benjamin and Sullivan’s model of marital change: relational changes observed among couples were influenced by increased relational resources and, to a lesser degree, gender consciousness, and in some cases this led to an increase in material resources/financial outcomes in the family. Their study in the United States and another application in Honduras (Murphy-Graham 2010) (both included only women) found the development of interpersonal skills and increased gender consciousness in women aided negotiation around change in communication and division of household work. Significant increases in gender consciousness, however, were not observed consistently in our study nor in the Honduran study (Murphy-Graham 2010). While rigidity around gender roles softened in some couples, with greater willingness to support each other, the only observed shifts in gender roles were around women working (in some couples).

The study findings point to areas for action in programming and policy. To start, the results highlight that mixed-sex approaches that engage both members of couples can be effective in facilitating positive change in relationships and reduction in IPV. While most couples in the sample did not attend together (apart from those who received CA support), there was nonetheless a pattern in which their separate involvement nurtured a reciprocal change process between them. These findings are important for the field of IPV prevention as there has been hesitation historically to engage and interview both members of a couple. The SASA! experience indicates that in the context of a community mobilisation intervention, it can be safe and effective if precautions are taken. Other studies on treatment for couples experiencing IPV and behavioural HIV prevention have also found engaging couples together can be more effective than single-gender approaches (El-Bassel and Wechsberg 2012; Stith et al. 2004).

The findings also reinforce the need for greater focus on promoting positive relationship values and dynamics in partner violence prevention, echoing recent calls in the literature for this (Bartholomew and Cobb 2010; Johnson 2010; Langhinrichsen-Rohling 2010). First, they indicate learning relationship skills can generate interaction patterns that encourage relationship growth and prevent conflicts from escalating to violence—even when other factors contributing to IPV remain constant (e.g. socioeconomic constraints). Interestingly, SASA! did not extensively promote relationship skills per se (the content of the Activist Kit focused more broadly on values and is purposely not prescriptive). Yet, from the examples in videos, dramas and CA support, couples clearly learned new ways to deal with relationship challenges. This suggests greater inclusion of relationship skills content may be beneficial in IPV prevention interventions. For example, helping couples resolve issues of fundamental disagreement peacefully—an ongoing challenge noted by many couples—could be helpful. To this end, there is an extensive relationship education field that the IPV prevention field may benefit from. It includes empirically supported relationship education tools (Halford 2011; Wadsworth and Markman 2012), some tailored to the specific challenges facing couples in low-income contexts (e.g. extreme financial strain) (Bradley and Gottman 2012).

Second, the data suggests by promoting relationship values and nurturing positive relationship dynamics, more balanced power can be achieved in relationships without necessarily addressing gender roles head on. This may be a softer and more effective way to achieve shifts in these areas without requiring individuals to overtly reject existing norms, addressing the challenges around backlash observed in some contexts (Wyrod 2008). While there has been growing evidence and debate in high-income contexts on the importance of relationship dynamics in partner violence (Capaldi et al. 2012; Ehrensaft et al. 2003), this is one of the only studies that examines this in an African setting.

Finally, the study makes a case for identifying the specific mechanisms through which positive change occurs either in terms of preventing violence or facilitating desistance and relational change. Examining desistance may suggest future avenues for research and yield insights with practical utility for guiding comprehensive prevention (Walker et al. 2013). Likewise, focusing on mechanisms identifies what must be retained or achieved through other means when taking a programme to scale or adapting it to another setting. Knowing what is most critical allows for programme adaptation when it is not feasible, cost effective or culturally appropriate to replicate a pilot programme exactly as designed.

## Limitations

The study’s small, purposive sample of people who reported relationship improvement limits the generalisability of its conclusions. While the findings illustrate that change is possible in established relationships with similar characteristics (e.g. ongoing relationship distress, rare to occasional physical violence), we cannot necessarily extrapolate to shorter relationships or those with more severe violence. For example, SASA! supports couples with severe violence differently (e.g. helping women to report violence and leave when appropriate) which may involve very different change processes. Despite our limited sample, the presence of similar themes in other qualitative work of SASA! (Kyegombe et al. 2014b) (albeit with individuals, not couples) suggests some of the changes observed in our study may be found beyond the sample. Third, the fact that eight couples were recruited through male RCT participants may have introduced a bias towards men who were more open to SASA!’s ideas. Change might be more complex for other couples, even though no discernible differences emerged from the two couples identified through the women.

Relying on a single interview represents another limitation of our study. Collecting data at multiple time points places less reliance on recall and allows the researcher to assess the consistency in participants’ accounts of change. The timeline tool, however, was a strength, improving recall. Perhaps the greatest threat to validity is the possibility that participants exaggerated the impact of SASA! on their lives, out of a desire to please the investigators and present the programme in a positive light. Interviewing partners separately and comparing their accounts helped address this limitation, as overlaps in the two narratives increased the validity and trustworthiness of the changes reported. Some of the reported change may also have evolved from other factors in the environment beyond SASA! that could have influenced social norms around relationships and marriage in Uganda (Nyanzi et al. 2005; Parikh 2007). Overall, future research may benefit from conducting interviews at multiple time points with a larger, more diverse sample of couples.

## Conclusion

This study draws attention to the value of working not only with women and men to prevent IPV, but explicitly with couples, within a broader community framework. Examining relationship trajectories from both partners’ perspectives throws into sharp relief how relationships can improve and violence wane. Key are interventions that generate hope and belief in alternative ways of being in relationship together with simple tools and support for change. The prevention field may benefit from community mobilisation approaches that include relationship skills and support for both women and men alongside interventions with local leaders and service providers.
